# Identification and validation of a prognostic signature based on six immune-related genes for colorectal cancer

**DOI:** 10.1007/s12672-024-01058-1

**Published:** 2024-05-28

**Authors:** Lifeng Zheng, Ziyu Xu, Wulou Zhang, Hao Lin, Yepeng Zhang, Shu Zhou, Zonghang Liu, Xi Gu

**Affiliations:** Department of General Surgery, Nanjing Jiangbei Hospital, Nanjing, Jiangsu China

**Keywords:** Colorectal cancer, Immune genes, Risk signature, Prognosis

## Abstract

**Background:**

Colorectal cancer (CRC) is a prevalent malignancy with high mortality and morbidity rates. Although the significant efficacy of immunotherapy is well established, it is only beneficial for a limited number of individuals with CRC.

**Methods:**

Differentially expressed immune-related genes (DE-IRGs) were retrieved from The Cancer Genome Atlas (TCGA), Gene Expression Omnibus (GEO), and ImmPort databases. A prognostic signature comprising DE-IRGs was developed using univariate, LASSO, and multivariate Cox regression analyses. A nomogram integrating the independent prognostic factors was also developed. CIBERSORT was used to assess immune cell infiltration (ICI). Furthermore, wound-healing, colony formation, migration, and invasion assays were performed to study the involvement of ACTG1 in CRC.

**Results:**

A signature including six DE-IRGs was developed. The overall survival (OS) rate was accurately estimated for TCGA and GSE38832 cohorts. The risk score (RS) of the signature was an independent factor for OS. Moreover, a nomogram encompassing age, RS, and pathological T stage accurately predicted the long-term OS probability of individuals with CRC. The high-risk group had an elevated proportion of patients treated with ICIs, including native B cells, relative to the low-risk group. Additionally, ACTG1 expression was upregulated, which supported the proliferation, migration, and invasion abilities of CRC cells.

**Conclusions:**

An immune-related prognostic signature was developed for predicting OS and for determining the immune status of individuals with CRC. The present study provides new insights into accurate immunotherapy for individuals with CRC. Moreover, ACTG1 may serve as a new immune biomarker.

**Supplementary Information:**

The online version contains supplementary material available at 10.1007/s12672-024-01058-1.

## Introduction

The incidence and death rates of colorectal cancer (CRC) rank second among all malignant tumors globally [1], and they are increasing annually in China [2]. CRC is a multifactorial disease with a genetic component, and unhealthy habits, such as alcohol abuse, smoking, physical inactivity, and obesity, are risk factors [3]. Surgery is commonly recommended for early-stage patients with CRC [4]. However, despite surgery, approximately 20% of patients with advanced CRC experience recurrence and metastasis [5, 6]. For such patients, neoadjuvant chemotherapy, radiotherapy, and immunotherapy have been used with significant therapeutic benefits. Nonetheless, these secondary treatments have side effects. Therefore, patients with CRC should undergo personalized systemic treatment regimens, which may improve quality of life and prolong survival.

Over the past decade, immunotherapy has been extensively explored and subsequently established as an effective therapy for CRC with microsatellite instability [7, 8]. Checkpoint inhibitors are an immunotherapeutic approach, and checkpoint inhibitors targeting programmed cell death 1 (PD1) or programmed cell death-ligand 1 (PD-L1) are typically recommended for mismatch repair-deficient metastatic CRC patients [9]. Recently, Cercek et al. reported that dostarlimab, a PD-1 inhibitor, is effective in treating mismatch repair-deficient, locally advanced rectal cancer. A previous study has reported that 12 patients receiving the single agent dostarlimab achieved a complete clinical response for at least 6 months [10], indicating that immunotherapy can lead to long-term remission in certain patients with CRC. Hence, precision immunotherapy can be used to investigate the molecular attributes of immunotherapy.

Recent studies have shown that immune-related genes (IRGs) regulate the immune system to promote or suppress tumor development [11, 12]. Prediction tools based on IRGs have been reported to reflect tumor progression and predict patient survival with better accuracy than existing tools [13, 14]. Such prediction tools can also aid in identifying effective treatments that can better balance side effects and survival benefits for patients with CRC. Wang et al. established an immune-linked prognostic signature with eight genes that depicts the dysregulated tumor immune microenvironment in CRC [15]. Similarly, Song et al. constructed a seven circRNA-related signature that assesses survival and reflects the immune status of individuals with CRC [16]. However, a reliable signature for CRC is rare, and most of the signatures that have been developed lack validation. Thus, the aim of the present study was to develop a better and more accurate signature than those previously reported.

## Materials and methods

### Data source

Gene expression profiles of six GEO datasets (GSE90524, GSE84983, GSE134525, GSE104364, GSE109454, and GSE115856) were obtained from the GPL16956 platform. Additionally, the GSE38832 chip data with survival time data were accessed at GEO (https://www.ncbi.nlm.gov/geo/). IRGs were derived utilizing ImmPort (http://www.immport.org). Additionally, the clinical information and transcriptomic data of 546 individuals with CRC were retrieved from TCGA (http://portal.gdc.cancer.gov). Furthermore, the protein expression data of six DE-IRGs were acquired from the human protein atlas (HPA) (http://www.proteinatlas.org/) database.

### Integration of microarray data and recognition of DE-IRGs

The major sources of bias and variability in integrating microarray data are heterogeneity and potential variables. Thus, to eliminate these biases, the data were integrated and batch-normalized with the help of the sva and limma packages in R software, respectively. The limma package was also used for the identification of DEGs. A log2 |fold change (FC) |> 1 and adjusted *P* < 0.05 were considered to indicate statistical significance. DE-IRGs were acquired by overlapping IRGs and DEGs, and they were visualized using the gplots package in R software.

### Bioinformatics analysis of DE-IRGs

Gene Ontology (GO) analysis and Kyoto Encyclopedia of Genes and Genomes (KEGG) analysis were performed using the org.Hs.eg.db and clusterProfiler packages in R software.

### Establishment and validation of the prognostic risk model

Following the exclusion of 42 individuals with an unsatisfactory follow-up period (less than 30 days), a total of 506 individuals from TCGA were screened for further analysis. The individuals were randomly classified into training (n = 253) and testing (n = 253) cohorts. The clinicopathological attributes of the included individuals are presented in Table 1. The training cohort was used to identify the DE-IRGs related to OS, and Univariate Cox regression analysis revealed prognostic DE-IRGs. LASSO-penalized regression analysis was applied to decrease the number of prognostic DE-IRGs using the glmnet package in R software. Finally, multivariate Cox regression was applied to screen the optimal prognostic DE-IRGs. The risk score (RS) of each individual was computed utilizing the coefficient and mRNA expression of prognostic DE-IRGs as follows: RS = ${\sum }_{i=1}^{N}(\text{Expi}*\text{Coei})$; where N is the gene number; Expi is the gene expression level; and Coei is the coefficient value.

**Table 1:** Clinical characteristics of 506 colorectal cancer patients

Variables	Group	Total cohort	Training cohort	Testing cohort	*P* value
Gender	Female	229	121	108	0.246
	Male	277	132	145	
Age	< 65	207	99	108	0.416
	≥ 65	299	154	145	
Stage	I	90	43	47	0.936
	II	187	97	90	
	III	140	67	73	
	IV	73	38	35	
	Unknown	16	8	8	
Vital status	Dead	92	45	47	0.818
	Alive	414	208	206	

The median of the RSs was selected as the cutoff threshold to segregate individuals into high- and low-risk groups. Survival analysis between the high- and low-risk groups was conducted using the survival and survminer packages in R software. To assess the accuracy of the risk model, receiver operating characteristic (ROC) analysis was performed to obtain the 1-, 3-, and 5-year area under the curve (AUC) values using the survivalROC package in R software. Additionally, the entire TCGA cohort, the testing cohort, and GSE38832 were utilized to validate the model. Univariate and multivariate Cox regression analyses aided in the evaluation of the independent prognostic value of the RS and clinical attributes in the entire TCGA cohort. Prognostic nomograms were generated using Cox regression coefficients with the rms package in R software. Calibration plots assessing the performance of these nomograms were generated using the regplot package in R software. ROC and decision curve analysis (DCA) curves were established to demonstrate the nomogram model's predictive capacity.

### Immune cell infiltration (ICI)

The tumor ICI was determined using the CIBERSORT algorithm based on the normalized gene expression data and the annotated gene signature matrix, outlining 22 immune cell subtypes. The data were retrieved from the CIBERSORT web portal (http://cibersort.stanford.edu/), and the perm was set to 1000. Samples with *P* < 0.05 were considered significant and utilized for subsequent investigation.

### Sample collection

A total of eight individuals with CRC, who were diagnosed with CRC by a professional pathologist, were selected from the Nanjing Jiangbei Hospital. The tumor and adjacent nontumor tissues were extracted via surgical resection, preserved in liquid nitrogen during transportation, and stored at − 80 °C until further analysis. This research was approved by the Ethics Committee of Nanjing Jiangbei Hospital. Informed consent was acquired from all the individuals involved.

### Real-time polymerase chain reaction (RT‒PCR) assay

TRIzol Reagent (Invitrogen, CA, USA) was used to extract total RNA from CRC tissues and adjacent nontumor tissues. A high-capacity cDNA reverse transcription kit (Thermo Fisher, USA) was used for reverse transcription of total RNA to complementary DNA (cDNA) strands. RT‒PCR was conducted using an LC480II (Roche, Switzerland) thermocycler and a SYBR Green kit (Thermo Fisher, USA). The primer pairs used for the six DE-IRGs are listed in Table S1. The 2^−△△Ct^ value was computed to determine gene expression levels, and glyceraldehyde-3-phosphate dehydrogenase served as a negative control.

### Cell culture and transient transfection

The SW480, SW620, HT29, and LOVO cell lines were incubated under 5% CO_2_ at 37 °C in Roswell Park Memorial Institute medium or Dulbecco’s modified Eagle’s medium (DMEM) (Gibco BRL, Rockville, Maryland, USA) supplemented with 10% fetal bovine serum. For transient transfection, SW480 and SW620 cells (1 × 10^5^ cells/ml) were seeded into 24-well plates prior to the day of transfection and cultured for 24 h to reach 60% confluence. The cells were transfected with short hairpin RNA (shRNA) against ACTG1 (Sangon Biotech, Shanghai, China) using Lipofectamine 2000 (Invitrogen, Carlsbad, USA) and subsequently incubated for 48 h. The target sequences for the shRNAs are as follows:

ACTG1-shRNA1, GCTGGCAAGAACCAGTTGTTT;

ACTG1-shRNA2, CCGAGCCGTGTTTCCTTCCAT;

ACTG1-shRNA3, CGCATCCTCCTCTTCTCTGGA.

### Colony formation and cell proliferation assays

SW480 and SW620 cells (5 × 10^4^ cells/well) were seeded into 96-well plates. The cells were transfected with NC shRNA or ACTG1-shRNA1, with six replicate wells for each group. At 24, 48, and 72 h posttransfection, 10 μl of 3-(4,5-dimethyl-2-thiazolyl)-2,5-diphenyl-2-H-tetrazolium bromide (MTT) solution was added to each well. The absorbance of each well was measured spectrophotometrically at 492 nm after incubation for 2 h. For the colony formation assay, SW480 and SW620 cells transfected with NC shRNA or ACTG1-shRNA1 were seeded into each well of a 6-well plate (500 cells/well). After 14 days, the colonies were fixed with methanol and stained with 0.1% crystal violet to facilitate counting the number of colonies. All assays were repeated three times.

### Cell invasion and migration assays

Matrigel-precoated Transwell inserts were placed into 24-well plates, and 600 μl of DMEM was added to each well. SW480 and SW620 cells (1 × 10^6^ cells) transfected with NC shRNA or ACTG1-shRNA1 were added to the upper chamber. Cells that traversed the membrane between the upper and lower chambers of the Transwell insert were fixed with methanol for 30 min and stained with 0.1% crystal violet. Wet cotton swabs were used to remove the cells that did not traverse the membrane. Finally, the plates were imaged in three different fields of view.

### Wound-healing assay

 SW480 and SW620 cells transfected with NC shRNA or ACTG1-shRNA1 were added to 6-well plates (1 × 10^6^ cells/well). A 1-mm wide scratch was then made across the cell layer using a sterile pipette tip. The suspended cells were discarded, and bright-field images were acquired for the control. The plates were imaged at 0 and 24 h after scratching the cell layer at an identical location, and the width of the scratch was also measured.

### Statistical analyses

All the statistical analyses were performed with R software (version 4.0.5). The Kaplan–Meier survival curves were compared utilizing the log-rank test. False discovery rate correction was applied to adjust the *P* value for multiple tests. The significance of gene expression across the normal and tumor tissues was assessed using the Student’s t test. Univariate and multivariate Cox regression analyses were performed to identify independent prognostic factors related to OS. *P* < 0.05 was considered to indicate statistical significance.

## Results

### Detection of DE-IRGs

**Fig. 1:**
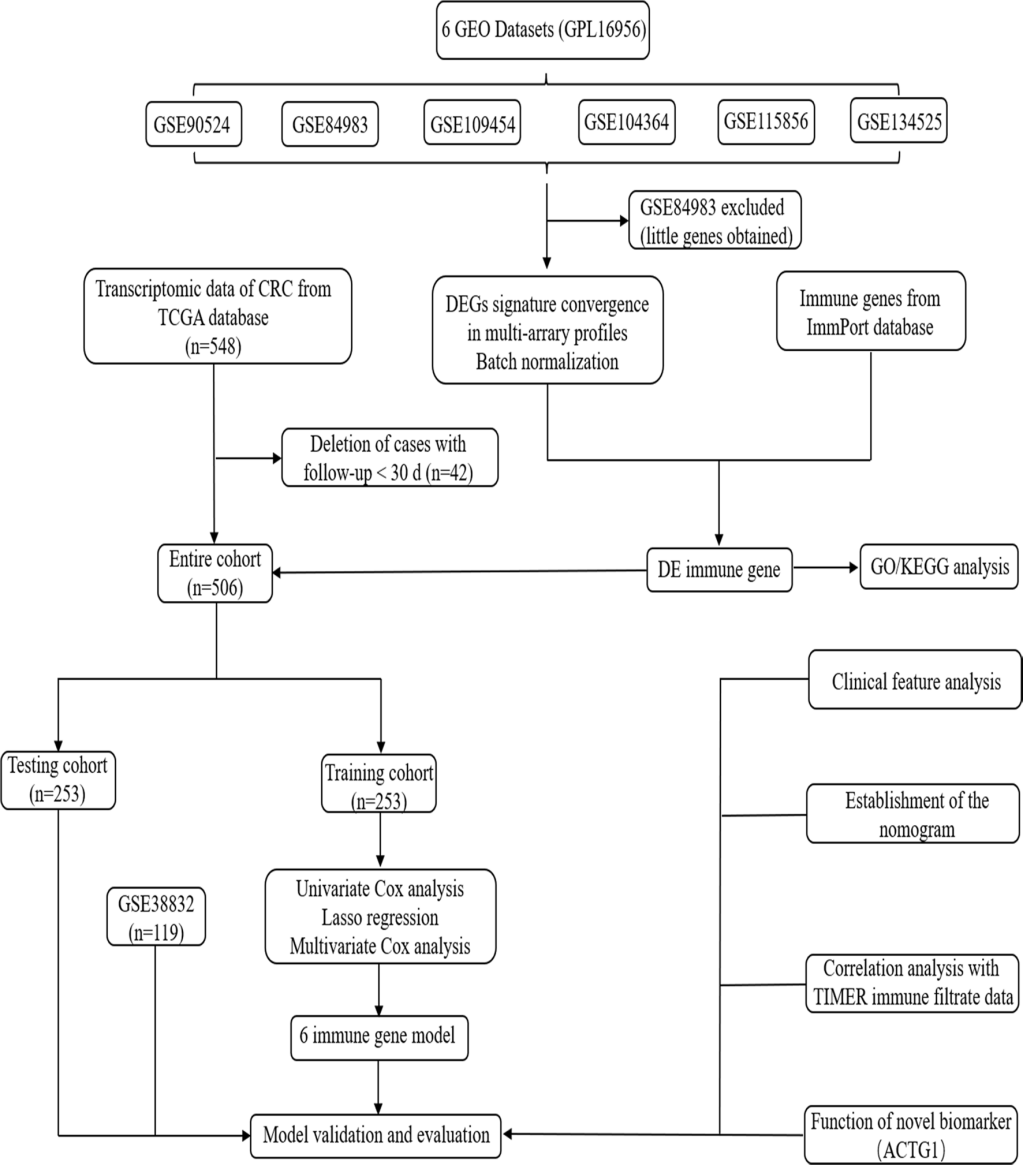
Flowchart of the study

**Fig. 2:**
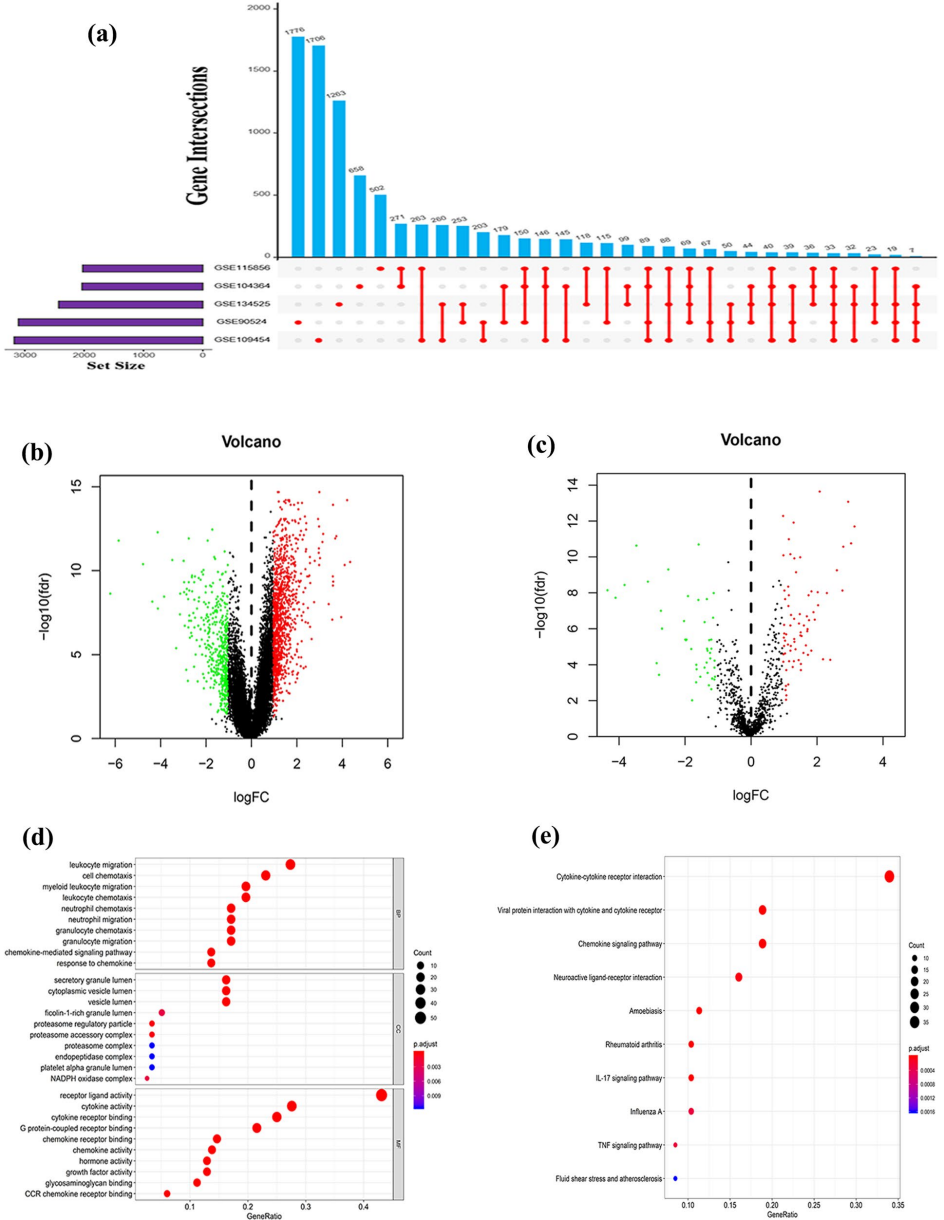
Identification and functional enrichment analysis of differentially expressed immune-related genes (DE-IRGs). **a** UpSet Venn diagrams of the DEGs identified in five GEO datasets. **b** Volcano plot of DEGs. **c** Volcano plot of DE-IRGs. **d** Gene Ontology (GO) analysis of DE-IRGs. **e** Kyoto Encyclopedia of Genes and Genomes (KEGG) pathway enrichment analysis of DE-IRGs

The present study design is shown by a flowchart in Fig. 1. Six datasets (GSE90524, GSE84983, GSE134525, GSE104364, GSE109454, and GSE115856) were downloaded, and their platform information was retrieved from GEO (Table S2). All the datasets had 32,386 genes after reannotation, except for the GSE84983 dataset, which had 2557 genes, indicating that this dataset was not consistent with the other datasets and was thus excluded due to the limited number of genes obtained. DEG analysis was performed for each dataset. DEGs between tumor and adjacent nontumor tissues were identified in the GSE90524, GSE134525, GSE104364, GSE109454, and GSE115856 datasets, with 3116, 2434, 2037, 3187, and 2029 DEGs, respectively, (Fig. 2a; Fig. S1a–e). Moreover, integrated analysis with a batch normalization method was performed to merge the five datasets into one dataset. A total of 1610 DEGs, including 1146 upregulated and 464 downregulated genes, were identified in the merged dataset (Fig. 2b). Overlap of the DEGs with the IRGs identified 118 DE-IRGs, including 73 upregulated and 45 downregulated genes (Fig. 2c). The top 10 upregulated and downregulated DE-IRGs are listed in Table S3.

### Functional enrichment analysis of DE-IRGs

GO and KEGG analyses helped to identify the functional roles of the DE-IRGs. GO analysis indicated that the most enriched terms of cellular components, biological processes, and molecular functions were secretory granule lumen, leukocyte migration, and receptor‒ligand activity, respectively. KEGG analysis indicated that the most significantly enriched pathway was cytokine‒cytokine receptor interaction. The top 10 most enriched terms identified by GO and KEGG analyses are shown in Fig. 2d, e.

### Establishment of the risk model

**Fig. 3:**
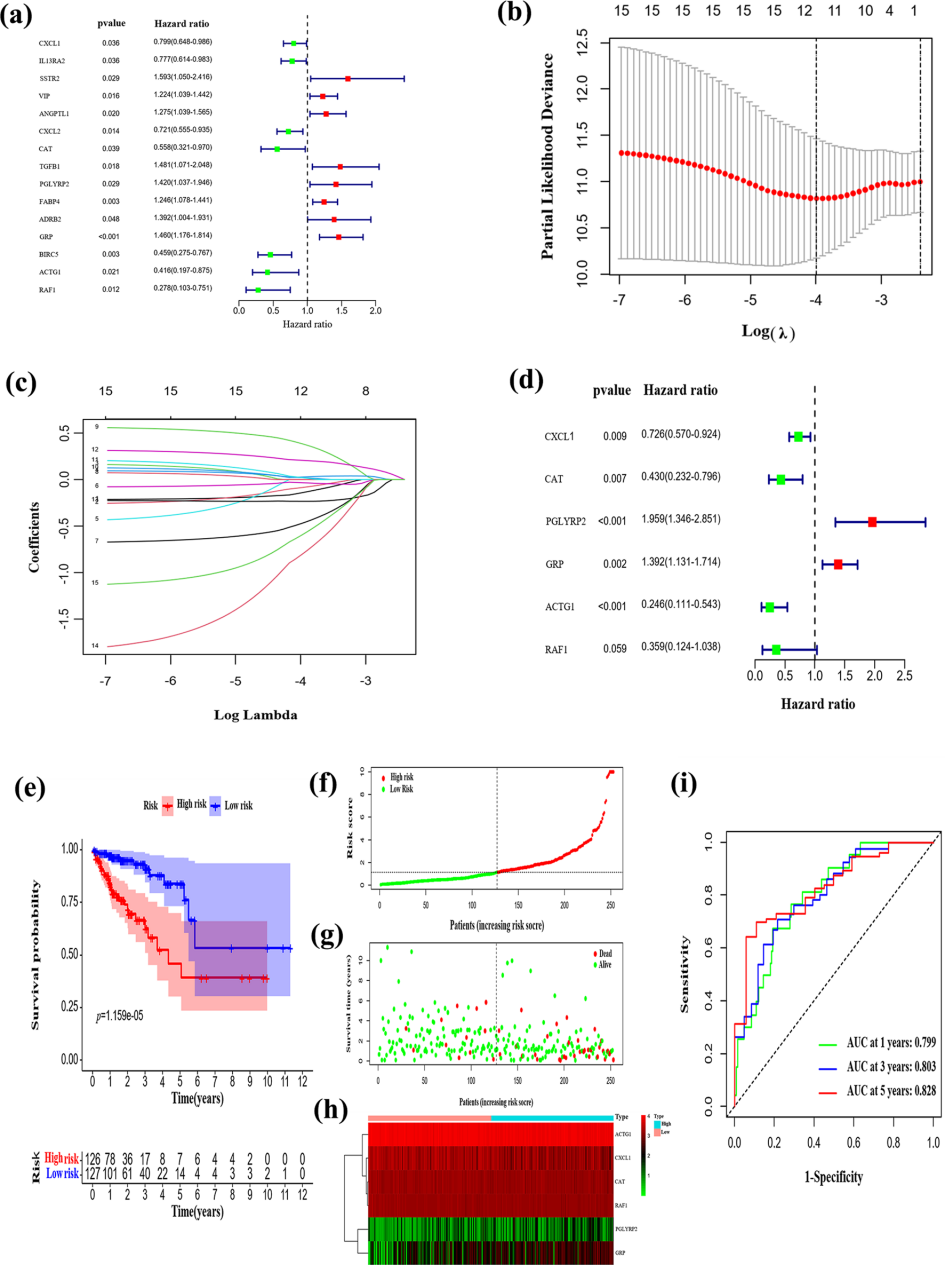
Establishment of the prognostic risk model based on the training cohort. **a** Fifteen differentially expressed immune-related genes (DE-IRGs) were identified by Univariate Cox regression analysis. **b** and **c** Twelve DE-IRGs were identified by LASSO-Cox regression analysis. **d** Six optimal DE-IRGs were identified for the establishment of the prognostic risk model by multivariate Cox regression analysis. **e**–**i** K‒M survival curves, risk score distributions, survival status scatter plots, heatmaps of the six DE-IRGs, and time-dependent receiver operating characteristic (ROC) curve analysis of the prognostic risk model in the training cohort

Univariate Cox regression analysis was used to identify the DE-IRGs related to OS. Using the training cohort, 15 DE-IRGs were obtained (Fig. 3a), which were reduced to 12 through LASSO-Cox regression analysis (Fig. 3b, c). After multivariate Cox regression, the following six optimal DE-IRGs were selected to develop the prognostic risk model: C–X–C motif chemokine ligand 1 (CXCL1); catalase (CAT); gastrin-releasing peptide (GRP); actin gamma 1 (ACTG1), raf-1 proto-oncogene, serine/threonine kinase (RAF1); and peptidoglycan recognition protein 2 (PGLYRP2) (Fig. 3d). The RS of each individual was computed with the following equation: RS = (− 0.32029*expression of CXCL1) + (− 0.84476*expression of CAT) + (0.67235*expression of PGLYRP2) + (0.33094*expression of GRP) + (− 1.4025*expression of ACTG1) + (− 1.0255*expression of RAF1).

Individuals in the training cohort were classified into high- (n = 126) and low-risk (n = 127) groups according to the median RS. The Kaplan–Meier survival curve indicated that high-risk individuals had poorer OS than low-risk individuals (*P* = 1.159e-05, Fig. 3e). The RS distribution curve, survival status plot, and heatmap of the risk groups are shown in Fig. 3f–h. The respective AUC values of the training cohort at 1, 3, and 5 years were 0.799, 0.803, and 0.828, respectively (Fig. 3i). These findings demonstrated the robust ability of the six-IRG-based model to predict the prognosis of individuals with CRC.

### Verification of the model

**Fig. 4:**
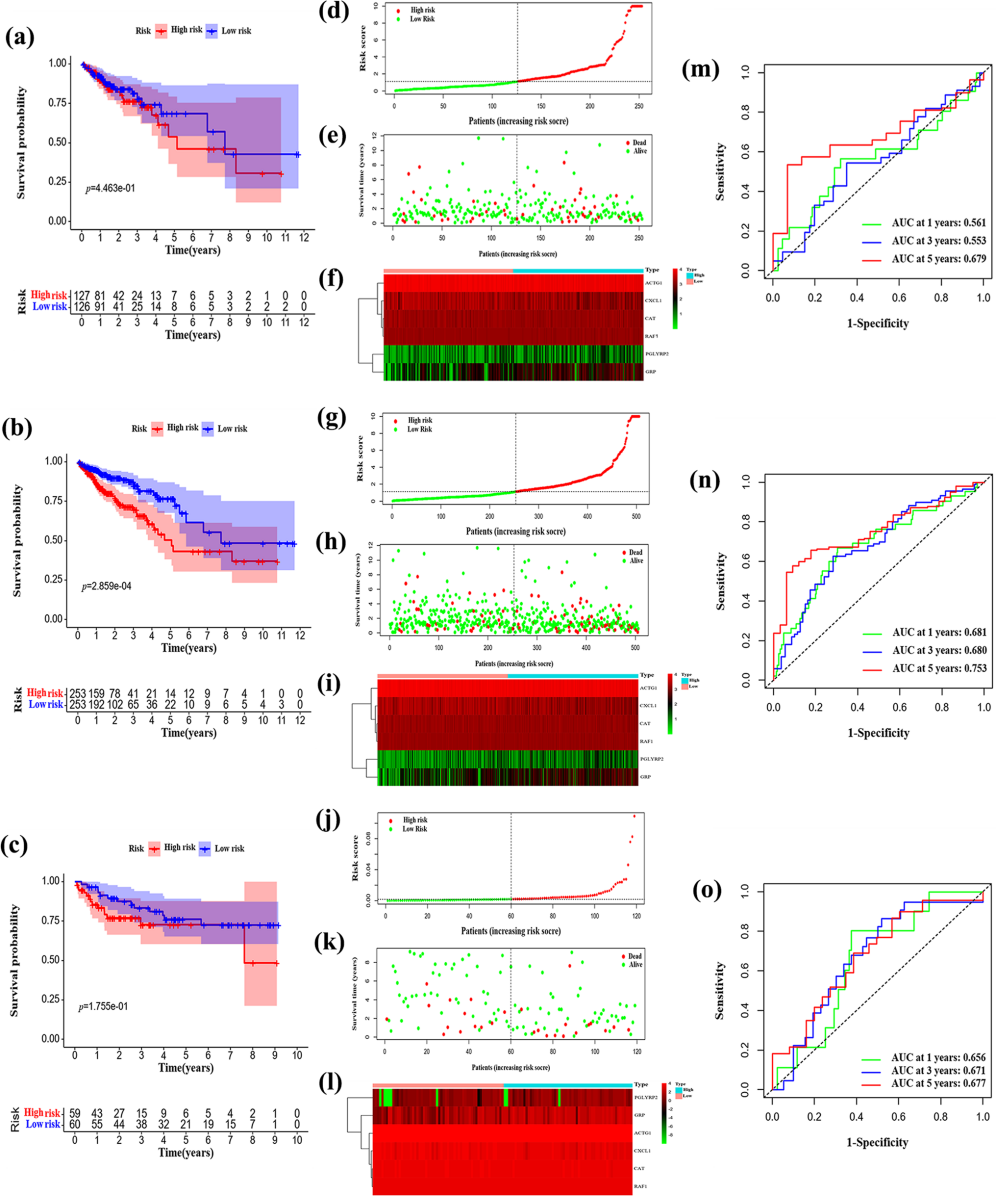
Validation of the prognostic risk model in the external cohorts. **a**–**c** Kaplan‒Meier survival curves of the prognostic risk model in the testing, TCGA, and GSE38832 cohorts. **d**–**l** Risk score distribution, survival status scatter plots, and heatmap of the six differentially expressed immune-related genes (DE-IRGs) of the prognostic risk model in the testing, TCGA, and GSE38832 cohorts. **m**–**o** Time-dependent receiver operating characteristic (ROC) curve analysis of the prognostic risk model in the testing, TCGA, and GSE38832 cohorts

To investigate the accuracy of the model, the RSs of each individual in the testing cohort, the entire TCGA cohort, and the GSE38832 cohort were computed. Individuals were classified into high- and low-risk groups according to the median value of the RS in each cohort. In the three validation cohorts, high-risk individuals had poorer OS than low-risk individuals (testing cohort: *P* = 4.463e−01, Fig. 4a; entire TCGA cohort: *P* = 2.859e−04, Fig. 4b; GSE38832: *P* = 1.755e−01, Fig. 4c). The RS distribution curve, survival status plot, and heatmap of the risk groups in the three validation cohorts are depicted in Fig. 4d–l. Mortality was greater in the high-risk group than in the other groups. Moreover, the gene expression profiles of the validation cohort were similar to those of the training cohort. In the testing cohort, the AUC values at 1, 3, and 5 years were 0.561, 0.553, and 0.679, respectively (Fig. 4m). For the entire TCGA cohort, the AUC values at 1, 3, and 5 years were 0.681, 0.680, and 0.753, respectively (Fig. 4n). For the GSE38832 dataset, the AUC values at 1, 3, and 5 years were 0.656, 0.671, and 0.677, respectively (Fig. 4o). Thus, the findings of the validation cohort were consistent with those of the training cohort, thereby demonstrating the predictive accuracy of the risk model.

### Prognostic evaluation and clinical utility of the model

**Fig. 5:**
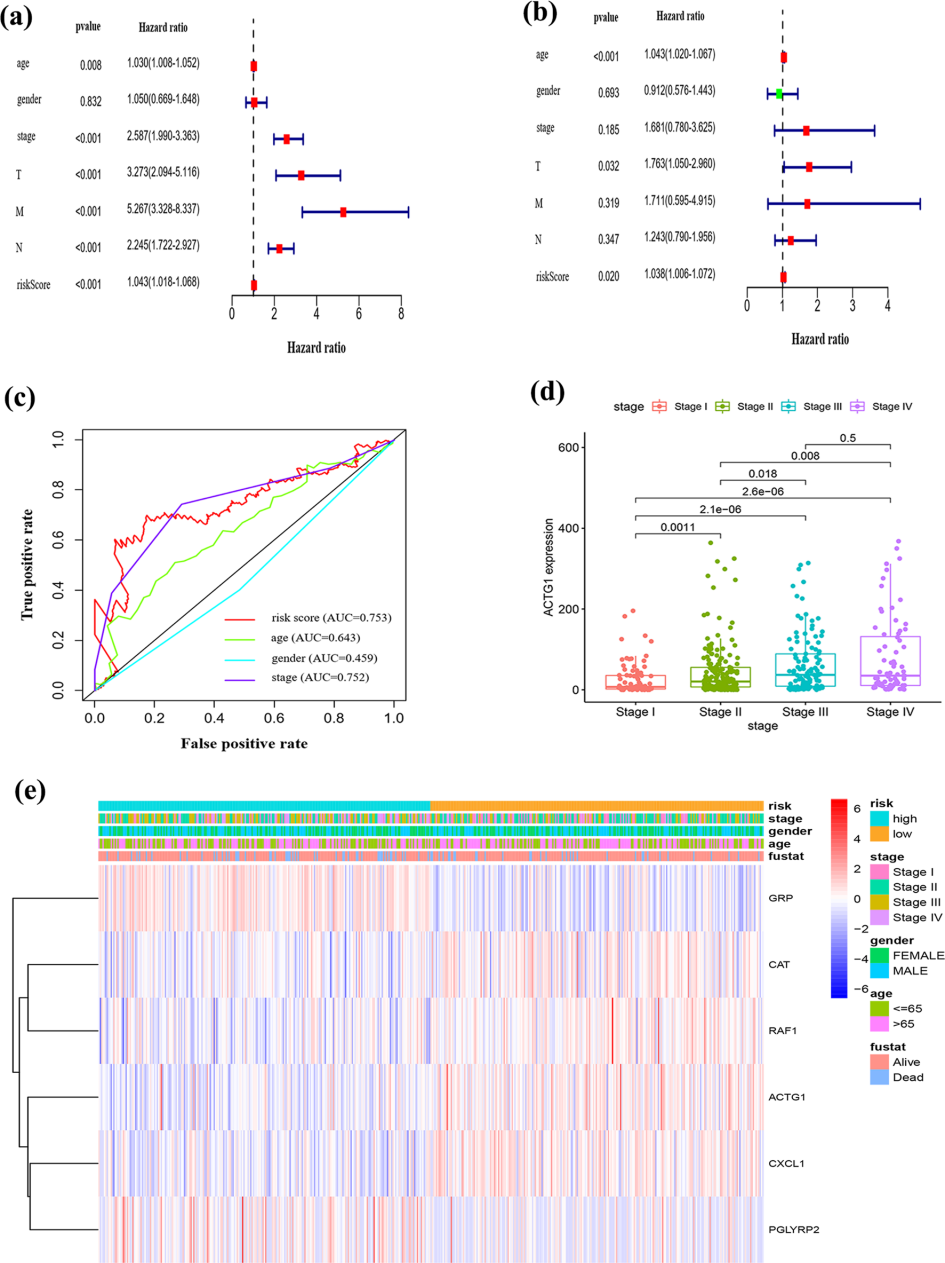
Independent prognostic value of the model in TCGA cohort. **a**–**c** Univariate and multivariate Cox regression analyses and time-dependent receiver operating characteristic (ROC) curve analysis of the risk score and clinical features at 5 years. **d** Relationship between ACTG1 expression and pathological stage. **e** Heatmap of the expression of six differentially expressed immune-related genes (DE-IRGs) and clinical features

Univariate and multivariate Cox regression analyses facilitated the determination of the prognostic value of the model (Fig. 5a, b). Age, pathological T stage, and the RS were considered as independent prognostic factors for OS in the entire TCGA cohort (*P* < 0.05). The respective AUC values of age, sex, stage, and RS at 5 years were 0.643, 0.459, 0.752, and 0.753, which implied that the RS was the most accurate for 5-year OS prediction (Fig. 5c). A comparison of the expression of the six genes with clinical features revealed that ACTG1 expression decreased with increasing TNM stage (*P* < 0.05, Fig. 5d), which suggested that ACTG1 may aid in early tumor diagnosis. The expression profiles of the genes are illustrated in Fig. 5e.

### Establishment of the prognostic nomogram

**Fig. 6:**
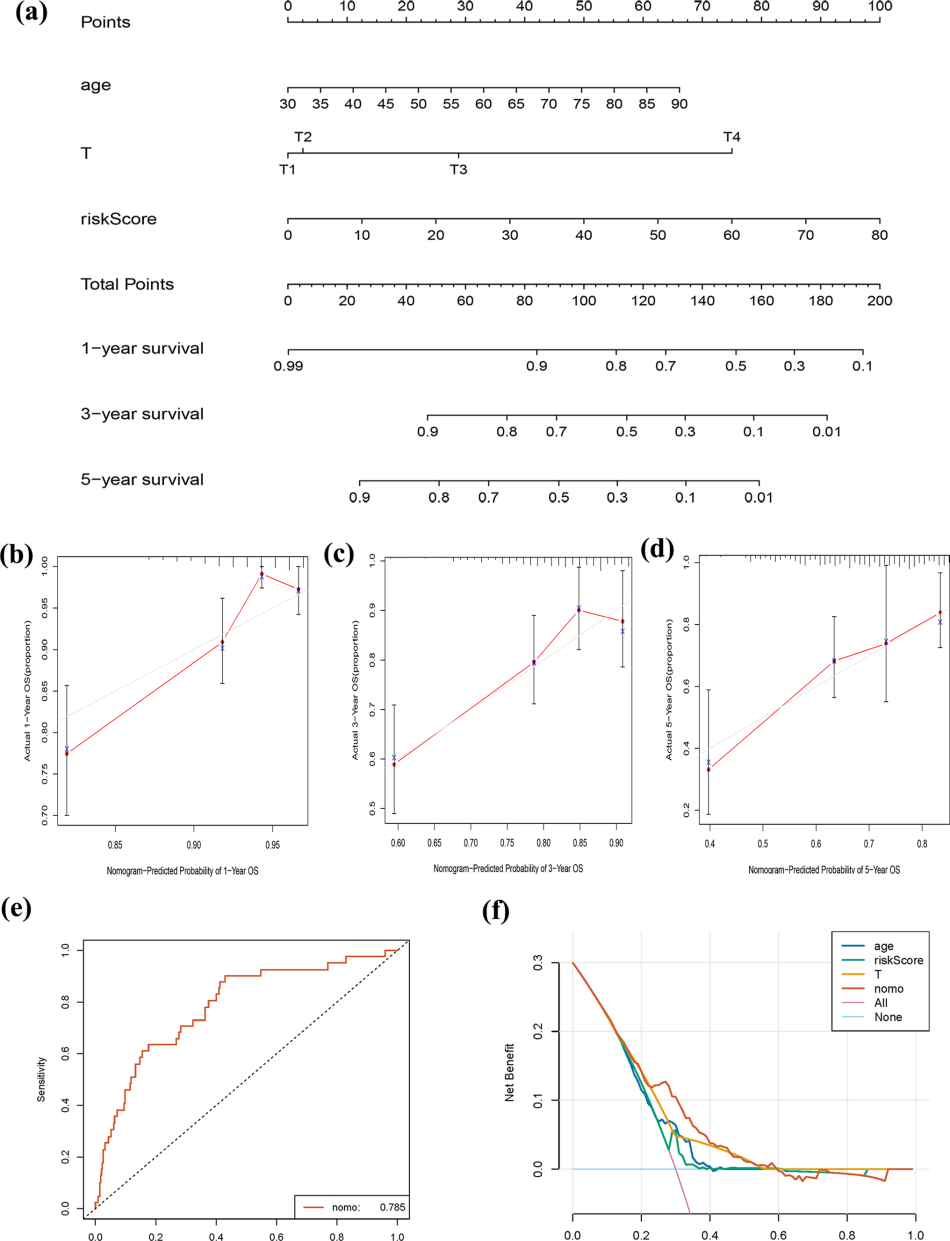
Nomogram for predicting the survival of patients with colorectal cancer (CRC). **a** Nomogram to predict the probability of 1-, 3-, and 5-year overall survival (OS) in CRC patients. **b**-**d** Normative curve for predicting the probability of 1-, 3-, and 5-year OS in CRC patients. **e**, **f** ROC and DCA curves of nomogram model

Independent prognostic factors, such as age, pathological T stage, and the RS, were used to develop the nomogram (Fig. 6a), which enabled the visualization of prognostic factors and aided in assessing the survival probability of individuals with CRC. The calibration plots for the 1-, 3-, and 5-year OS predictions are illustrated in Fig. 6b–d. The corrected curve was closer to the ideal curve, which indicated strong coherence between the predicted and observed values. The ROC of nomogram model was 0.785 (Fig. 6e). The prediction effect of the DCA curve suggested that the performance of nomogram model was better than the others (Fig. 6f).

### Relationship of ICI efficacy with the risk model

**Fig. 7:**
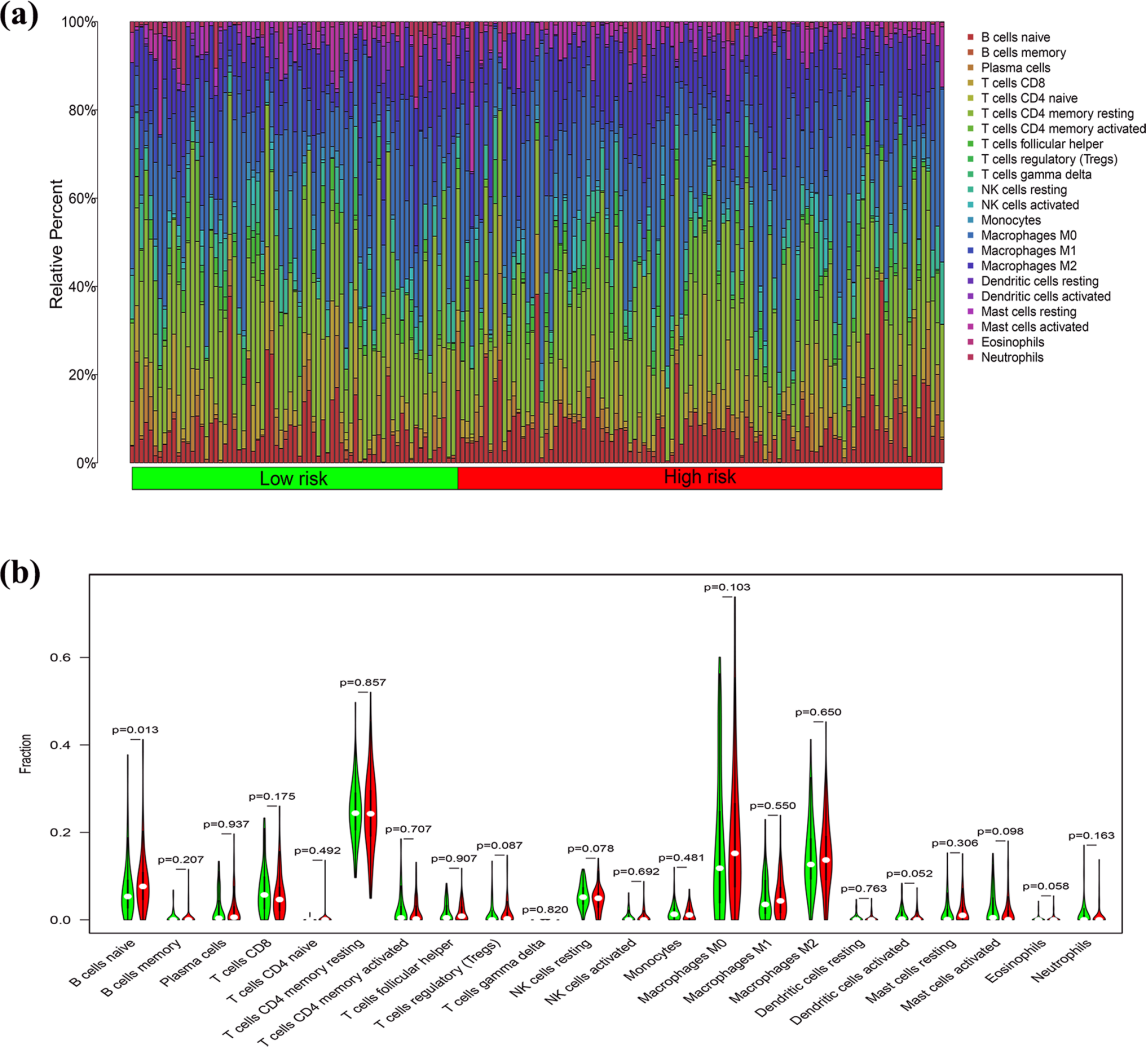
Immune infiltration status. **a** Infiltration ratio of 22 immune cell types in the high- and low-risk group samples. **b** Differences in immune cell types between the high- and low-risk groups

The proportions of 22 immune cells in every sample were computed using the CIBERSORT algorithm, resulting in 232 samples with *P* > 0.05 for subsequent analyses. The composition of the immune cell population varied across samples and between different groups (Fig. 7a). A comparison of the ICI levels across the high- and low-risk groups revealed that naive B cells were enriched in the high-risk group (*P* = 0.013, Fig. 7b).

### Validation of the expression of the six genes in the model

The HPA database was used to explore the protein expression of the six genes. CXCL1 and GRP were excluded because they were not detected in the HPA. Among the genes, ACTG1, CAT, and RAF1 were upregulated in tumor tissues relative to normal tissues. However, PGLYRP2 was downregulated in tumor tissues (Fig. S2a). To further examine the expression of the six genes, RT‒PCR was performed on eight pairs of samples. All genes, except PGLYRP2, were upregulated in tumor tissues, with significant increases found for CAT (*P* < 0.01), CXCL1 (*P* < 0.05), and RAF1 (*P* < 0.05) (Fig. S2b–g), which agreed with the previous results.

### Knockout of ACTG1 suppresses the proliferation, migration, and invasion of CRC cells

**Fig. 8:**
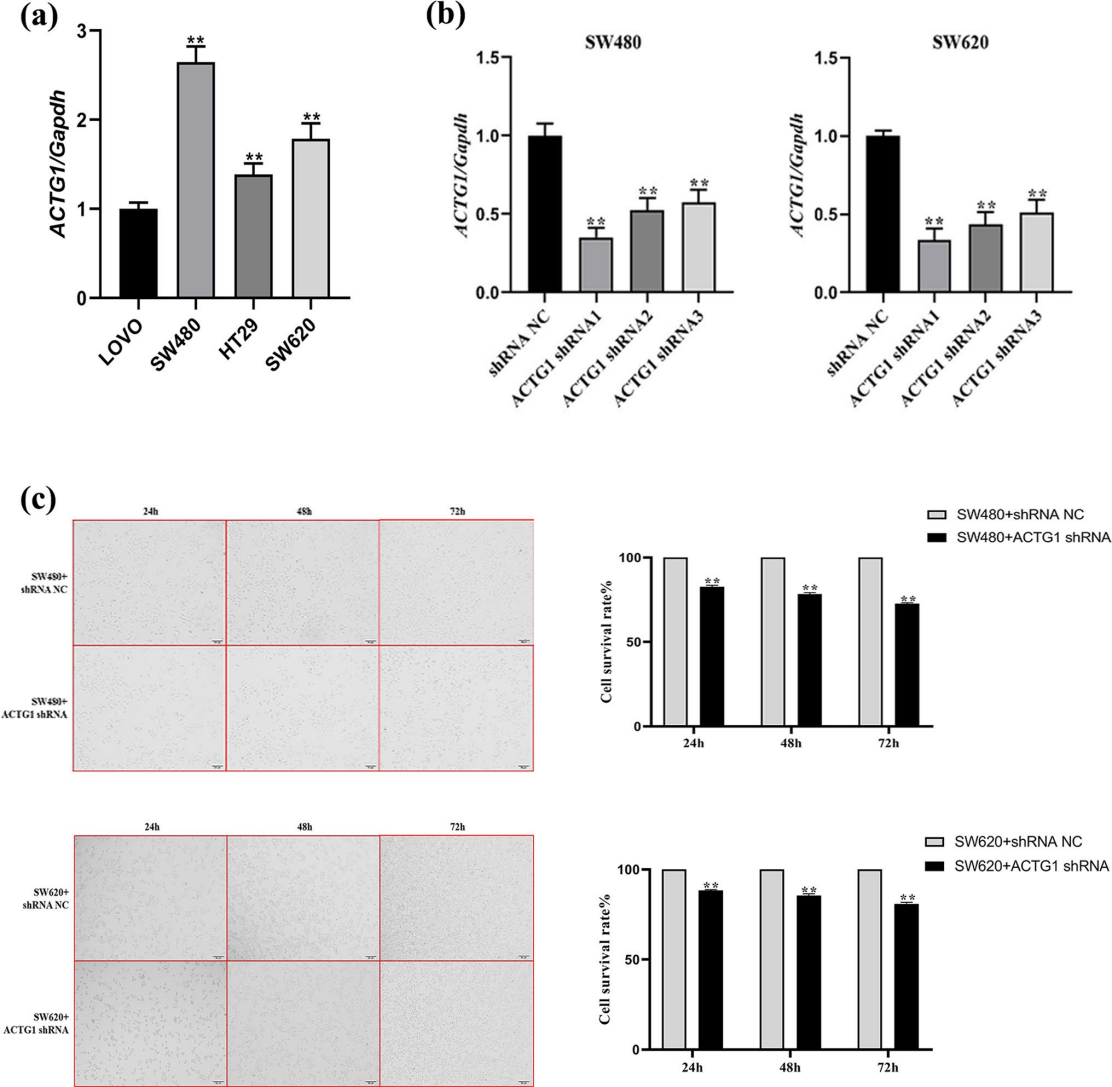
ACTG1 knockdown inhibits cell proliferation in vitro. **a** ACTG1 expression was upregulated in colorectal cancer (CRC) cells. **b** ACTG1 expression in ACTG1-shRNA SW480 and SW620 cells. **c** Growth curves of SW480 and SW620 cells after transfection with ACTG1 shRNA were determined by MTT analysis. **P < 0.01

**Fig. 9:**
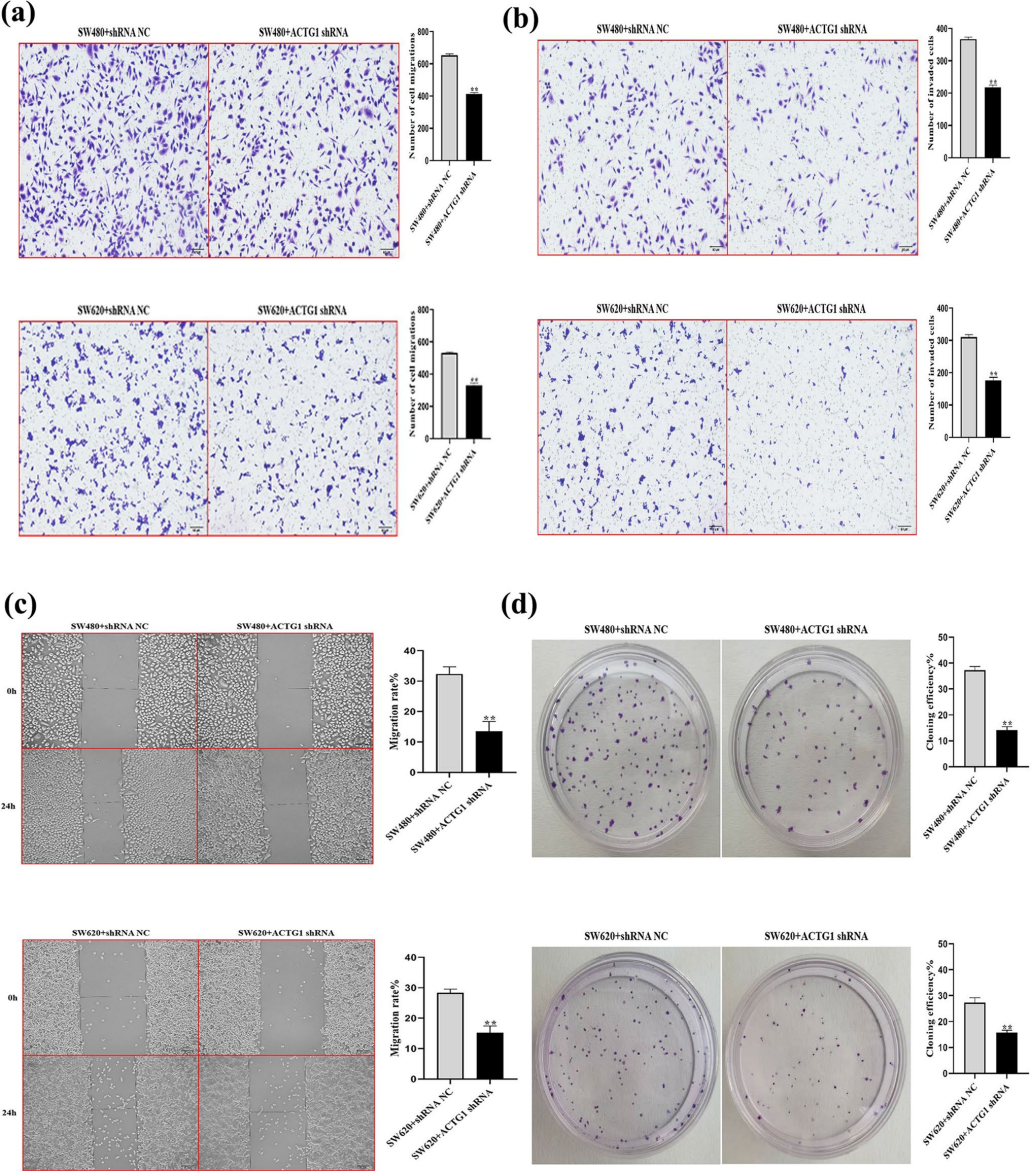
ACTG1 knockdown inhibits cell migration and invasion in vitro. **a**–**c** Decreased cell migration and invasion in ACTG1-deficient SW480 and SW620 cells. **d** Clonogenic assay of ACTG1-deficient SW480 and SW620 cell lines showing decreased colony formation. **P < 0.01

To investigate the involvement of ACTG1 in the progression of CRC, MTT, wound-healing, colony formation, migration, and invasion assays were performed. Because ACTG1 expression was upregulated in the SW480 and SW620 cell lines (Fig. 8a), these cell lines were selected for further investigation. After knocking down ACTG1 (Fig. 8b), the proliferation of the cells was inhibited according to the MTT assay (Fig. 8c). CRC cell migration and invasion were also significantly suppressed by downregulating ACTG1 expression (Fig. 9a–c). In addition, ACTG1 promoted CRC cell colony formation (Fig. 9d). Thus, these results suggested that high ACTG1 expression indicates poor outcomes for individuals with CRC.

## Discussion

Approximately 590,000 new CRC cases and 310,000 CRC-related deaths were reported in China in 2022 [2]. To date, approximately half of patients with CRC die due to recurrence and metastasis, suggesting the need for a precise signature and novel biomarkers for CRC.

In the present study, five GEO datasets were integrated and batch-normalized into one dataset, revealing 1610 DEGs in the merged dataset. After overlapping with the immune genes, 118 DE-IRGs were obtained. The DE-IRGs were strongly linked to leukocyte migration and cytokine‒cytokine receptor interactions, suggesting that they are involved in tumor development and metastasis. Finally, an immune-related prognostic signature comprising six DE-IRGs (ACTG1, CAT, CXCL1, GRP, RAF1, and PGLYRP2) was developed.

As an actin protein, ACTG1 contributes to cell division, migration, chromatin remodeling, vesicle transport, and other cellular functions [17]. ACTG1 encodes the γ-actin, which serves an important function in nonmuscle cells, and it is also abundant in the auditory hair cells of the cochlea [18]. Furthermore, ACTG1 acts as a tumor promoter in different cancers, such as skin cancer, hepatocellular cancer, and lung cancer [19–21]. Liu et al. reported that ACTG1 acts as a target gene of miR-10a in CRC and is highly expressed in CRC cells [22]. However, the mechanism of action of ACTG1 in CRC carcinogenesis warrants further research.

CAT is the key antioxidant enzyme that decomposes H_2_O_2_ and counteracts the damaging effects of reactive oxygen species (ROS) [23]. The overproduction of ROS can lead to redox imbalance and cause tumor initiation and progression [24]. Justyna et al. reported that the activity of CAT in individuals with CRC is lower than that in control individuals, and the expression of CAT can predict lymph node metastasis [25]. Moreover, Adam et al. studied the immunoexpression of CAT in colorectal lesions, and they reported that elevated expression levels of CAT in adenoma and decreased expression of CAT in adenocarcinoma lead to the development of CRC [26]. Notably, Reyhaneh et al. demonstrated that curcumin inhibits tumor growth by enhancing the activity of CAT in colitis-related CRC [27].

Chemokines are a group of soluble chemotactic cytokines and a key element of inflammation. As inflammation is a hallmark of cancer, chemokines have been shown to promote tumorigenesis via various pathways [28]. According to the position of key cysteine residues, chemokines are classified into four classes (CXC, CX3C, CC, and C) [29]. CXCL1 belongs to the C–X–C family of chemokines and interacts with CXCR2 [30, 31]. The function of CXCL1 in CRC has been widely researched. Ogata et al. noted that CXCL1 expression is linked to the degree of CRC differentiation and can predict the prognosis of individuals with CRC [32]. Similarly, Gong et al. reported that CXCL1 is upregulated in tumor tissues, and they demonstrated that it is a diagnostic marker for colon cancer based on analysis of one TCGA cohort and two Guangxi cohorts [33]. Additionally, Zhuang et al. reported that miR-145-5p inhibits colon cancer development by suppressing the expression of CXCL1 and ITGA2 [34].

GRP is the mammalian counterpart of bombesin and induces gastrin secretion from G cells in the gastric antrum [35]. GRP is involved in the mitogenesis of cancer cells, and it also has paracrine and endocrine effects [36]. Recently, many studies have focused on the function of GRP and its receptors in CRC. Li et al. reported that GRP can predict the prognosis of individuals with colon cancer with high sensitivity and specificity [37]. Additionally, GRP receptors have also been demonstrated to serve as biomarkers for early CRC diagnosis and colorectal tumor growth monitoring [38].

Raf-1, also called C-Raf, is part of the Raf family of protein kinases and is involved in the EGFR/Raf/MEK/ERK signaling pathway. The EGFR pathway is a classic antitumor therapeutic target that transmits extracellular signals into the nucleus through cell membrane receptors, participating in cell proliferation, differentiation, and other functions [39, 40]. Inhibitors of Raf-1 have been widely studied and promoted for the treatment of CRC. For instance, regorafenib, a Raf-1 inhibitor, has been approved for the oral treatment of metastatic CRC refractory to standard chemotherapy [41–43].

PGLYRP2 is a pattern recognition receptor and encodes a peptidoglycan recognition protein. PGLYRP2 is primarily expressed in the liver and participates in the regulation of innate immunity and immunosurveillance [44]. Moreover, PGLYRP2 gene polymorphisms are also associated with Parkinson’s disease [45, 46]. PGLYRP2 also acts as a biomarker for an adequate immune response against hepatocellular carcinoma and improves patient outcomes [47].

In the present study, PGLYRP2 was downregulated and recognized as a protective gene, whereas ACTG1, CAT, CXCL1, GRP, and RAF1 were upregulated and linked to poor survival. Moreover, ACTG1 expression increased with increasing TNM stage. To further evaluate the involvement of ACTG1 in CRC, ACTG1 expression in SW480 and SW620 cells was suppressed, which reduced the proliferation, migration, and invasion abilities of these cells. These results indicated that ACTG1 plays an active role in CRC carcinogenesis. Nevertheless, the precise involvement of the six IRGs in CRC progression warrants further investigation.

In the training cohort, individuals in the low-risk group had better OS compared to those in the high-risk group. ROC analysis revealed that the prediction of 5-year OS performed better than the prediction of 1-year or 3-year OS. The results were validated in the external cohorts and were consistent with those in the training cohort. In addition, the RS provided better predictive value in terms of accuracy than pathological stage, sex, and age. RS was established as an independent risk factor for predicting the OS of individuals with CRC. Moreover, RS and clinicopathological features aided the development of a nomogram, which confirmed the stability of the RS and the accuracy of its ability to predict survival. In conclusion, the developed model and nomogram may be useful for assessing the OS of individuals with CRC.

Immune cells in the tumor immune microenvironment contribute significantly to the progression of tumors, and the extent of ICI therapy has been demonstrated to influence the survival of individuals with CRC [48]. Additionally, the proportions of 22 immune cell types in each sample were evaluated, which demonstrated that naive B cells were significantly greater in the high-risk group than in the low-risk group. Naive B cells are white blood cells that have not been activated by an antigen, and they are involved in manufacturing antibodies, assisting in eliminating harmful pathogens, and preventing subsequent assaults from reoccurring. Yan et al. evaluated B cell immunity in CRC cells, and they reported attenuated antigen presentation and diminished antitumor immune capacity of CD40 + and CD27 + B cells in tumor tissues [49]. Overall, the degree of ICI treatment reflects the immune status of individuals with CRC, which may lead to differences in survival outcomes among risk groups.

The present study had several limitations. First, the number of samples was limited because they were retrieved from public databases. Second, the signature was obtained from a retrospective study, indicating the need for verification by prospective clinical trials. Finally, additional basic experiments should be conducted to investigate the molecular mechanisms of the signature.

## Conclusions

A six-IRG risk model was developed to predict OS and depict the immune status of individuals with CRC. Furthermore, ACTG1 was highlighted as a possible new immune biomarker in CRC. Overall, these findings aid in personalized treatment decisions and provide new immunotherapeutic measures for individuals with CRC.

### Supplementary Information

Additional file 1.

Additional file 2.

## Data Availability

Data is provided within the manuscript or supplementary information files. The GEO datasets can be downloaded from https://www.ncbi.nlm.gov/geo/. TCGA datasets and clinical data can be downloaded from http://portal.gdc.cancer.gov. The protein expression data can be downloaded from http://www.proteinatlas.org/.

## Abbreviations

CRC: Colorectal cancer
DE-IRGs: Differentially expressed immune-related genes
TCGA: The Cancer Genome Atlas
GEO: Gene Expression Omnibus
ICI: Immune cell infiltration
OS: Overall survival
RS: Risk score
PD1: Programmed cell death 1
PD-L1: Programmed cell death-ligand 1
IRGs: Immune-related genes
HPA: Human protein atlas
FC: Fold change
GO: Gene Ontology
KEGG: Kyoto Encyclopedia of Genes and Genomes
ROC: Receiver operating characteristic
AUC: Area under the curve
DCA: Decision curve analysis
RT‒PCR: Real-time polymerase chain reaction
cDNA: Complementary DNA
DMEM: Dulbecco’s modified Eagle’s medium
shRNA: Short hairpin RNA
MTT: 3-(4,5-Dimethyl-2-thiazolyl)-2,5-diphenyl-2-H-tetrazolium bromide
CXCL1: C–X–C motif chemokine ligand 1
CAT: Catalase
GRP: Gastrin-releasing peptide
ACTG1: Actin gamma 1
RAF1: Raf-1 proto-oncogene, serine/threonine kinase
PGLYRP2: Peptidoglycan recognition protein 2
ROS: Reactive oxygen species
