# Supplementary material for: Identification and validation of a prognostic signature based on six immune-related genes for colorectal cancer
Source: Discov Oncol. 2024 May 28;15:192. doi: 10.1007/s12672-024-01058-1 (PMC11133253; doi:10.1007/s12672-024-01058-1)
Supplement: Supplementary file 1 — Additional file 1. [file 12672_2024_1058_MOESM1_ESM.docx]

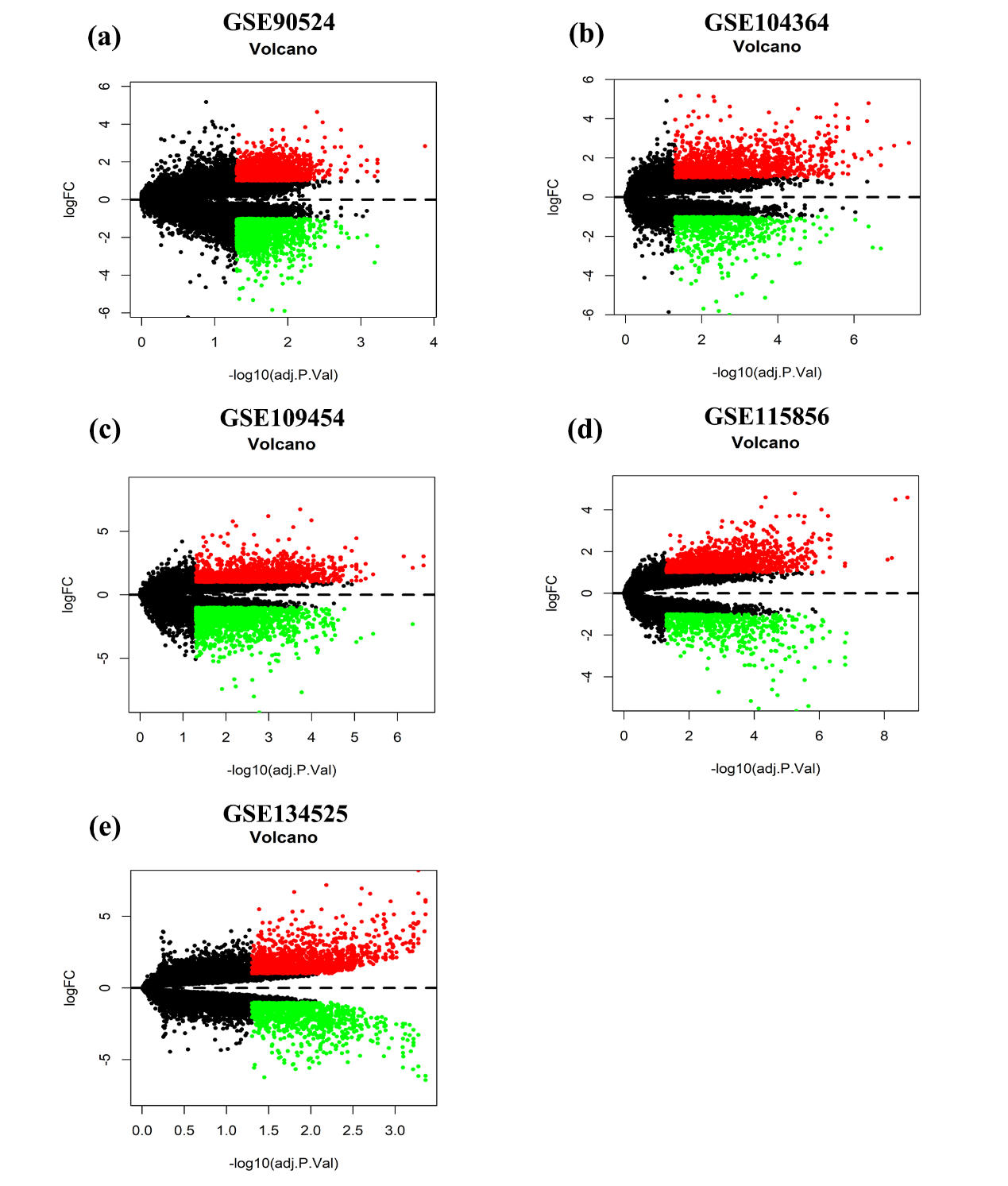


Figure S1: Differentially expressed genes (DEGs) in five GEO datasets. a-e Volcano plots of DEGs in the GSE90524, GSE104364, GSE109454, GSE115856, and GSE134525 datasets.


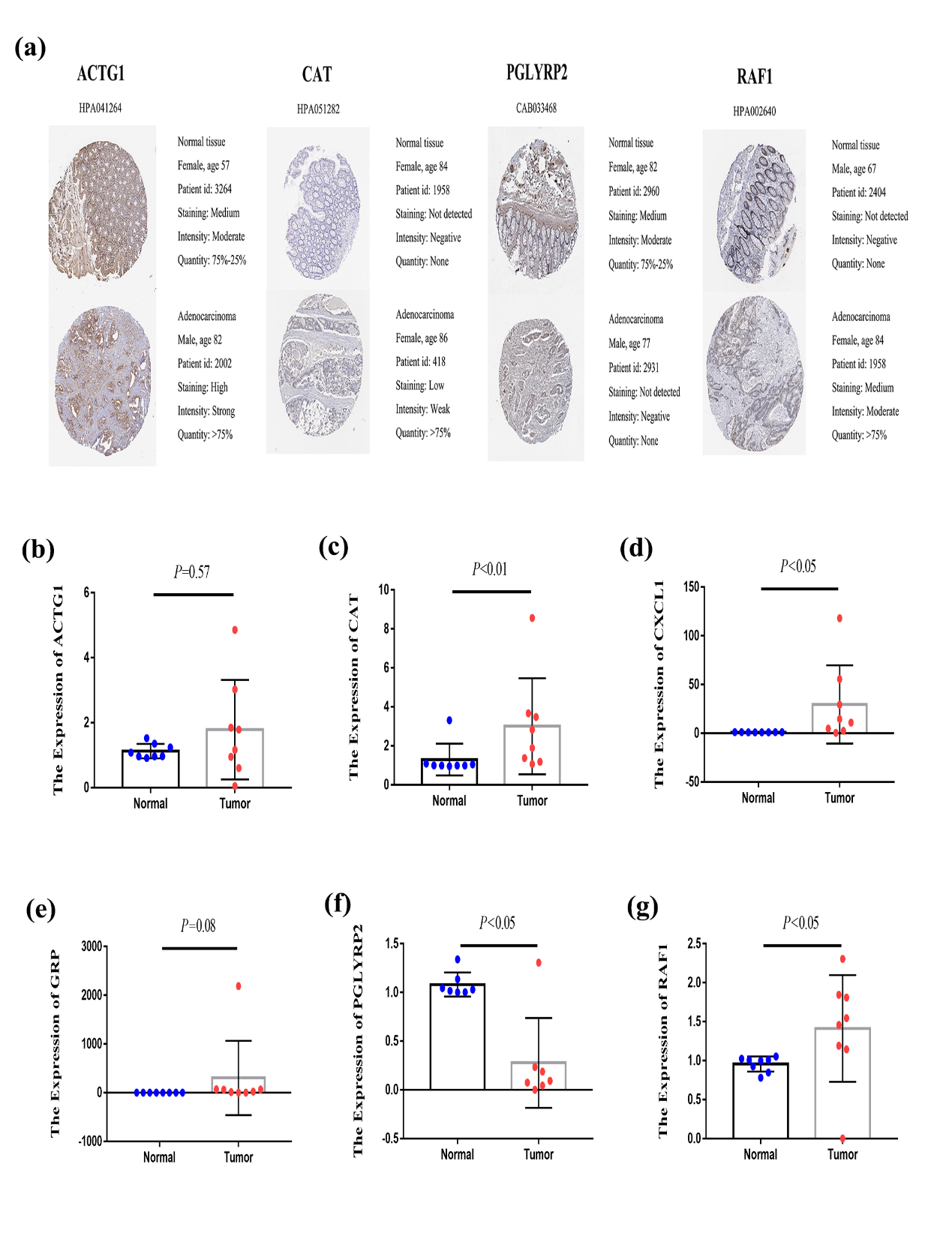
Figure S2: Validation of the expression of six selected genes in colorectal cancer (CRC) samples. a Representative protein expression of six selected genes in CRC tissue and normal tissue. The data were obtained from the Human Protein Atlas. b-g mRNA expression of six selected genes in eight paired CRC tissues and normal tissues.
