# Supplementary material for: Identification and validation of a prognostic signature based on six immune-related genes for colorectal cancer
Source: Discov Oncol. 2024 May 28;15:192. doi: 10.1007/s12672-024-01058-1 (PMC11133253; doi:10.1007/s12672-024-01058-1)
Supplement: Supplementary file 2 — Additional file 2. [file 12672_2024_1058_MOESM2_ESM.docx]

| Gene | Primer | Sequence (5’- 3’) |
| --- | --- | --- |
| ACTG1 | F | CTGAATTCTACGGCTTGGACTTTC |
|  | R | GTGGATCCTTTGCTGCATGGGTTA |
| CXCL1 | F | CCCAAGAACATCCAAAGTGTG |
|  | R | CATTCTTGAGTGTGGCTATGAC |
| CAT | F | CCAGAAGAAAGCGGTCAAGAA |
|  | R | GAGATCCGGACTGCACAAAG |
| GRP | F | GAGAACAGAAACCACCAGC |
|  | R | AGAGTCTACCAACTTTGCCT |
| PGLYRP2 | F | CATCGGCTACAGTTTCGTG |
|  | R | TGTAGTTGCCCACTATGGC |
| RAF1 | F | GGGAGCTTGGAAGACGATCAG |
|  | R | ACACGGATAGTGTTGCTTGTC |

**Table S1:** Primer Sequences used for real-time polymerase chain reaction (RT-PCR) assay

**Table S2**: Details of datasets from Gene Expression Omnibus (GEO) database

| Series accession | Platform | Tumor samples | Normal samples |
| --- | --- | --- | --- |
| GSE90524 | GPL16956 | 3 | 3 |
| GSE84983 | GPL16956 | 3 | 3 |
| GSE134525 | GPL16956 | 3 | 3 |
| GSE104364 | GPL16956 | 6 | 6 |
| GSE109454 | GPL16956 | 6 | 6 |
| GSE115856 | GPL16956 | 15 | 15 |

| Expression level | Gene symbol | LogFC | *p*Value | FDR |
| --- | --- | --- | --- | --- |
| Up | GDF15 | 3.165391 | 7.93E-15 | 2.01E-12 |
|  | LGR5 | 3.060863 | 1.25E-13 | 1.80E-11 |
|  | HSP90AB1 | 2.972978 | 1.03E-16 | 8.50E-14 |
|  | CST4 | 2.825816 | 2.13E-13 | 2.69E-11 |
|  | CXCL3 | 2.79966 | 2.18E-10 | 7.65E-09 |
|  | MMP12 | 2.630628 | 9.27E-12 | 5.66E-10 |
|  | SPP1 | 2.42968 | 6.08E-06 | 5.28E-05 |
|  | ULBP2 | 2.327852 | 3.10E-10 | 1.02E-08 |
|  | PPBP | 2.210363 | 5.67E-06 | 4.97E-05 |
|  | MET | 2.108873 | 1.86E-17 | 2.25E-14 |
| Down | GUCA2A | -4.32214 | 2.00E-10 | 7.19E-09 |
|  | GCG | -4.07569 | 6.25E-10 | 1.88E-08 |
|  | SST | -3.80286 | 9.07E-11 | 3.64E-09 |
|  | CCL23 | -3.44466 | 1.79E-13 | 2.34E-11 |
|  | ANGPTL1 | -3.09526 | 5.43E-11 | 2.41E-09 |
|  | FABP4 | -2.82972 | 9.91E-06 | 8.10E-05 |
|  | INSL5 | -2.75878 | 5.71E-05 | 0.000365 |
|  | CHP2 | -2.70192 | 4.48E-09 | 1.01E-07 |
|  | CCL19 | -2.66775 | 6.23E-08 | 9.94E-07 |

**Table S3:** Top 10 upregulated and downregulated differentially expressed immune-related genes (DE-IRGs)

FC: Fold change; FDR: False discovery rate
